# Improved intravenous lentiviral gene therapy based on endothelial-specific promoter-driven factor VIII expression for hemophilia A

**DOI:** 10.1186/s10020-023-00680-z

**Published:** 2023-06-12

**Authors:** Jie Gong, Rui Yang, Min Zhou, Lung-Ji Chang

**Affiliations:** 1 Chengdu Women’s and Children’s Central Hospital, School of Medicine, University of Electronic Science and Technology of China, Chengdu, 611731 China; 2 School of Medicine, University of Electronic Science and Technology of China, Chengdu, 610054 China; 3 Shenzhen Geno-Immune Medical Institute, 6 Yuexing 2nd Rd., 2nd Floor, Nanshan Dist., Shenzhen, 518057 Guangdong Province China

**Keywords:** Lentiviral vector, Gene therapy, Hemophilia A, Factor VIII, Endothelial-specific

## Abstract

**Background:**

Hemophilia A (HA) is an X-linked monogenic disorder caused by deficiency of the factor VIII (*FVIII*) gene in the intrinsic coagulation cascade. The current protein replacement therapy (PRT) of HA has many limitations including short term effectiveness, high cost, and life-time treatment requirement. Gene therapy has become a promising treatment for HA. Orthotopic functional FVIII biosynthesis is critical to its coagulation activities.

**Methods:**

To investigate targeted FVIII expression, we developed a series of advanced lentiviral vectors (LVs) carrying either a universal promoter (EF1α) or a variety of tissue-specific promoters, including endothelial-specific (VEC), endothelial and epithelial-specific (KDR), and megakaryocyte-specific (Gp and ITGA) promoters.

**Results:**

To examine tissue specificity, the expression of a B-domain deleted human *F8* (*F8BDD*) gene was tested in human endothelial and megakaryocytic cell lines. Functional assays demonstrated FVIII activities of LV-VEC-*F8BDD* and LV-ITGA-*F8BDD* in the therapeutic range in transduced endothelial and megakaryocytic cells, respectively. In *F8* knockout mice (*F8* KO mice, *F8*^*null*^ mice), intravenous (iv) injection of LVs illustrated different degrees of phenotypic correction as well as anti-FVIII immune response for the different vectors. The iv delivery of LV-VEC-*F8BDD* and LV-Gp-*F8BDD* achieved 80% and 15% therapeutic FVIII activities over 180 days, respectively. Different from the other LV constructs, the LV-VEC-*F8BDD* displayed a low FVIII inhibitory response in the treated *F8*^*null*^ mice.

**Conclusions:**

The LV-VEC-*F8BDD* exhibited high LV packaging and delivery efficiencies, with endothelial specificity and low immunogenicity in the *F8*^*null*^ mice, thus has a great potential for clinical applications.

**Supplementary Information:**

The online version contains supplementary material available at 10.1186/s10020-023-00680-z.

## Background

HA is an X-linked recessive disorder that results in serious bleeding after injury, or in severe cases, spontaneous bleeding. This disorder is caused by a single gene mutation in the *F8* gene that participates in the hemostasis (Nathwani et al. 2017). Existing protein replacement therapy (including human FVIII concentrate, porcine FVIII concentrate, recombinant FVIII concentrate and so on) improves the patient's quality of life, but does not cure the disease. Gene therapy has reported promising results in the correction of underlying deficiencies in HA. Adeno-associated virus vector (AAV) gene therapy for HA has moved from human trials to a final product (Valoctocogene Roxaparvovec) by European Commission (Ozelo et al. 2022; High et al. 2018; Nathwani et al. 2018; Rangarajan et al. 2017; Pasi et al. 2020). On the other hand, since Powell et al. conducted the first intravenous (iv) oncoretroviral HA gene therapy, there has been no further report of in vivo patient trial of oncoretroviral or LV HA gene therapy (Powell et al. 2003). Nevertheless, clinical trials based on LVs are in active pursue. While gene therapy for HA has been explored for decades, many obstacles remain in achieving optimal *F8* gene therapy efficacy (Herzog 2015). One of the major limitations in HA gene therapy is the inhibitor formation mainly due to antibody formation. Besides, the natural exposure of AAV in humans presents a major limitation in AAV-based gene therapy, yet this has not been extensively investigated in LV-based gene therapy studies.

Many studies indicate that FVIII is mainly synthesized in the liver. This makes hepatocyte an ideal target for HA gene therapy and this tissue site may help establish immune tolerance towards ectopic FVIII expression (Greig et al. 2017). Recent studies reveal that functional FVIII or Factor IX may be expressed by cell types including muscle cells (Arruda et al. 2017), endothelial cells (ECs) (Kren et al. 2007; Wang et al. 2016; Merlin et al. 2017), myoblasts and fibroblasts (Lee et al. 2004), and these cells may be alternative targets for gene therapy of hemophilia. Importantly, some have shown that tissue-specific *F8* gene expression may alleviate the adverse inhibitor problem i*n vivo* (Merlin et al. 2017; Kuether et al. 2012). Several tissue-specific promoters have been tested to direct the expression of FVIII protein in specified tissue types. For examples, megakaryocyte-specific promoters have been tested to express FVIII protein in hematopoietic stem cells (HSCs) gene transfer (Ide et al. 2007; Du et al. 2013). The endothelial system is part of the hemopoiesis system and the expression of FVIII protein in situ can be readily secreted into blood. Therefore, endothelial-specific expression based on a VEC promoter, which drives the expression of a late-stage marker of the ECs, has also been explored (Olgasi et al. 2018). Further, the expression of functional FVIII protein has been confirmed in ECs (Merlin et al. 2017; Olgasi et al. 2018; Gao et al. 2019; Rose et al. 2020).

Here we investigated universal versus tissue-specific promoters engineered in a LV system to explore the tissue-specific expression of *F8BDD*. The tissue-specific promoters included a vascular endothelial growth factor (VEGF) receptor promoter VEC, a synthetic early-stage endothelial cell specific promoter KDR, a megakaryocyte-specific promoter ITGA, and a late-stage megakaryocyte-specific (platelet-specific) promoter Gp1bα (Gp). In comparison with a strong universal EF1α promoter, the gene transfer efficiencies, FVIII protein functionality, and in vivo immunogenicity of these different LV promoters were extensively evaluated to support future clinical applications.

## Methods

### LV production

LVs were generated using the pEGWI LV system as previously described (Gong et al. 2021; Chang et al. 1999). *F8BDD* construct was created by ligation of the human *F8* (*hF8*) cDNA into the viral vector based on optimized nucleotide sequences between the A2 and A3 domains (Doering et al. 2002). *F8BDD* cDNA was cloned into the LV behind the human EF1α, VEC, KDR, Gp and ITGA promoters. The LVs were produced and concentrated as described previously (Chen et al. 2004; Chang 2010).

### Cell culture

The EA-hy926, DAMI, K562 and Raji cell lines were obtained from American Type Culture Collection, and cultured in DMEM or RPMI (Hyclone; Logan, Utah, USA), supplemented with 10% FBS, and 1% penicillin–streptomycin at 37 °C in 5% CO_2_ incubators.

### LV transduction

LV transduction was performed by incubating approximately 3 × 10^4^ EA-hy926 cells at MOI = 200 and 1 × 10^5^ DAMI, K562, or Raji cells at MOI = 100 with the various LVs in a final volume of 600 uL in a 6-well plate, supplemented with 8 µg/mL polybrene (Sigma-Aldrich; Saint Louis, MO, USA).

### Mouse protocols

Male wild-type (WT) and *F8*^*null*^ mice with C57BL/6J background were used in all experiments. The *F8*^*null*^ mice were purchased from Biosubstrate Technologies (Beijing, CN). Five- to Six-week-old mice were conditioned with non-myeloablative 600 cGy irradiation using an X-ray irradiation (Faxitron, Tucson, AZ, USA). The LVs injection was performed via tail vein injection four days after irradiation. Prebleeding was performed by tail-clipping followed by electrocautery. After the vector injection, the blood (20 μL) was collected in 3.5 μL citrate (Bioleaper, Shanghai, China) by retro-orbital bleeding procedure. Blood was taken by the heart puncture when mice were sacrificed. Plasma was frozen immediately and stored at − 80 ℃ until use. The mice were sacrificed 180 days after transplantation, and the whole blood collected by retro-orbital vein or heart puncture. The organs were harvested and frozen in − 80 ℃ until use.

### Analysis of LV-F8 RNA expression

The RNA was harvested from transduced cells using an RNA purification kit (Promega Corp. Madison, WI, USA). Approximately 200 ng of RNA was reverse transcribed into cDNA using a two-step HiScript III RT SuperMix kit (Vazyme, Nanjing, Jiangsu, CN). RT-PCR was performed at 37 ℃ for 15 min, and 85 ℃ for 5 s. The specific primers for *hF8* and human *GAPDH* were used for RT-PCR as previously reported (Gong et al. 2021). The electrophoresis gel was exposed and analyzed using a ChemiDoc Touch imaging system (Bio-Rad, Hercules, CA, USA).

### Quantitative analysis of human FVIII protein

The concentration of FVIII protein in the supernatants was determined using a human FVIII ELISA kit (Abcam, Cambridge, Cambs, UK), as per manufacturer’s instruction. Samples were diluted 1:100 in sample diluent from the kit and analyzed in duplicates. Standard curves of FVIII were generated based on the dilution instruction in the kit, and the optical density was read using a Cytation Hybrid Multi-Mode Reader (BioTek, Winooski, VT, USA). The unit for FVIII protein was shown as IU/mL.

### FVIII activity assays

The FVIII activity was measured based on activated Partial Thrombo-plastin Time (aPTT) assay, the two-step coagulation assay (chromogenic assay, HYPHEN BioMed, FR) or tail clip assay. The plasma was collected in PBS after centrifugation at 900*g* for 15 min. Dade Actin activated Cephaloplastin Reagent was purchased from Siemens, GER. In vivo clotting time per aPTT was capped at 5 min. Standard curves of chromogenic assay were generated using a normal pooled citrated WT plasma. The results were expressed as percentage of correction and analyzed by comparing LV-treated *F8*^*null*^ mice with that of WT and untreated mice. The tail clip assay was performed with modifications from a previously described protocol (Liu et al. 2012; Merlin et al. 2019). The mouse tail was immersed in 37 ℃ saline for 10 min to standardize the local blood circulation. The entire distal portion of the tail was cut off (diameter, about 2 mm) from anesthetized mice 120 days after transplantation. The time to cessation of blood flow was recorded. For those mice that bleeding never stopped, 10 min was set as the cutoff time.

### Detection of anti-F8 antibodies (Abs)

The activities of the inhibitory Abs were determined using the modified Bethesda method based on plasma samples from recipients 60 days after iv injection. Following incubation at 37 °C for 2 h, the residual FVIII activity was determined based on the chromogenic assay. One Bethesda unit was defined as the reciprocal of the dilution of test plasma at which 50% of hF8 activity was inhibited. The sensitivity of the assay was 1 Bethesda unit/mL.

### Flow cytometry

The EA-hy926 or DAMI cells transduced with LV with green fluorescence protein (LV-GFP) were resuspended in PBS buffer. The bone marrow (BM) cells were isolated from marrow cavities of femurs and tibiae of LV-treated mice. The liver cells were isolated from mice by collagenase digestion of liver as previously described (Follenzi et al. 2008). The spleens and lymph nodes were homogenized and single cell suspensions were prepared after erythrocyte lysis in red blood cell lysis buffer (BD Biosciences, Heidelberg, Germany). For analysis of cell-surface marker expression by flow cytometry, we incubated BM, liver or spleen cells in FACS buffer. Cells from control *F8*^*null*^ mice without LV transduction were used as controls. For intracellular staining, the cells were fixed in 0.5 mL fixation buffer (BioLegend, San Diego, CA, USA) in the dark at room temperature for 20 min, resuspended in the intracellular staining Perm Wash Buffer (BioLegend, San Diego, CA, USA), and centrifuged at 350*g* for 5–10 min. The antibodies used for surface and intracellular staining were as follows: APC anti-mouse CD34 (clone MEC14.7), PE-Cy7 anti-rat CD11b (clone M1/70), PE-Cy7 anti-mouse Ly6G (clone RB6-8C5), PE anti-mouse CD41 (clone MWReg30) and anti-F8 (1:500, sc-73597, Santa Cruz Biotechnology) Abs. The staining used the following Abs: anti-Rat F4/80 mAb (eBioscience) and anti-Rabbit CD31 mAb (Invitrogen). The secondary and the isotype-control Abs used to determine non-specific background signals were Alexa Fluor 561 or 637 goat anti-Rat lgG (H + L), Alexa Fluor 561 or 637 goat anti-mouse lgG (H + L), and Alexa Fluor 561 or 637 goat anti-Rabbit lgG (H + L) (Invitrogen). Cells were analyzed in a NovoCyte Quanteon flow cytometer (ACEA Biosciences, Palo Alto, CA, USA) and data were processed using the ACEA NovoExpress software.

### Statistical analysis

Statistical analysis was performed with the GraphPad Prism 8 software (GraphPad Inc., La Jolla, CA, USA). All data were presented as mean ± standard error of mean (SEM). Parameter test was carried out for data measurement conforming to normal distribution. When the data group was larger than 3, one-way Welch ANOVA test was used. If the variance is equal, the post hoc test used the Turkey test; if the variance is not equal, the post hoc selected the Games-Howell test. Kruskal–Wallis nonparametric test was performed for those that did not conform to normal distribution. Statistical significance was assumed for *p < 0.05; **p < 0.01; ***p < 0.001; ****p < 0.0001; n.s., no significant difference.

## Results

### LV construction and packaging analysis

**Fig. 1:**
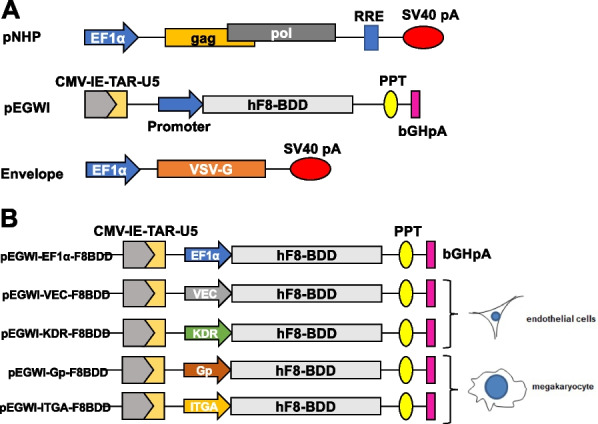
The NHP/TYF *F8BDD* LV system with an universal promoter and tissue-specific promoters for ECs and megakaryocytes. **A** Schematic illustration of self-inactivating LVs encoding the partially sequence-optimized human *F8BDD* gene. **B** Schematic illustration of the LV-*F8BDD* under the control of different promoters as depicted

**Fig. 2:**
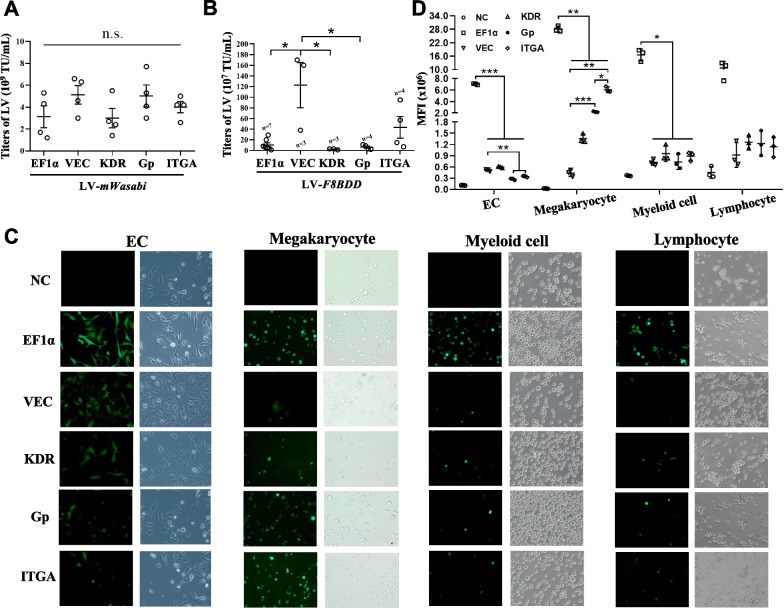
Lentiviral expression in endothelial, megakaryocyte, myeloid and lymphoid cells. **A**, **B** The titration of LV-*mWasabi* (n = 4) (**A**) and LV-*F8BDD* (n = 3 to 7) (**B**) constructs containing the five promoters (EF1α, VEC, KDR, Gp and ITGA) illustrating the packaging efficiencies. **C** GFP expression in transduced ECs, megakaryocytes, myeloid and lymphoid cells detected under a fluorescent microscope. The left panels represent green fluorescent signals and the right panels were under bright field, at 20 × magnification. **D** Quantitative analysis based on mean fluorescence intensity (MFI) by flow cytometry showing GFP expression in ECs, megakaryocytes, myeloid and lymphoid cells (n = 3). The differences in characteristics between groups were analyzed using the one way Welch ANOVA tests with Games-Howell post hoc tests (**A**, **D**), and Kruskal–Wallis tests (**B**); *p < 0.05, **p < 0.01, ***p < 0.001, n.s., no significant difference

The NHP/EGWI LV system is illustrated in Fig. 1A. LVs were constructed to express either the *F8BDD* gene or a reporter GFP gene (*mWasabi*) under five different promoters: universal EF1α, endothelial VEC and KDR, and megakaryocyte Gp and ITGA promoters (Fig. 1B). The packaging efficiency and titration of the different LVs were determined by quantitative PCR (qPCR) based on the integrated LV genomes, and the results showed that high titer LV-*mWasabi* at 1–6 × 10^9^ TU/mL could be produced (Fig. 2A). The LV titers for EF1α-, VEC-, KDR-, Gp- and ITGA-*F8BDD* constructs were (10.4 ± 10.2), (122.7 ± 73.5), (2.3 ± 1.2), (6.3 ± 4.4) and (43.5 ± 40.7) × 10^7^ TU/mL, respectively, with consistent 10- to 100-fold higher packaging efficiency for LV-VEC-*F8BDD* than the other vectors (Fig. 2B).

### Expression and functional analyses of LV-*F8BDD* in ECs and megakaryocytes

To examine tissue specificity, we first tested these LVs in different cell types including EA-hy926 (EC), DAMI (megakaryocyte), K562 (myeloid cell) and Raji (lymphoid cell). These cells were transduced with LV-*mWasabi* carrying different promoters at similar multiplicities of infection (MOI), and the vector copy number (VCN) was determined by qPCR using genomic DNA (gDNA) harvested from the transduced cells (Additional file 2: Table S1). We examined the stably transduced cells under a fluorescent microscope ten days after LV transduction. The result showed that EF1α and VEC promoters exhibited high, KDR promoter exhibited medium, and Gp and ITGA promoters exhibited low green fluorescence in ECs (Fig. 2C). On the other hand, in megakaryocytes, we observed high fluorescence signal for EF1α and ITGA promoters, medium signal for Gp promoter, and low signal for VEC and KDR promoters (Fig. 2C). In myeloid and lymphoid cells, only the EF1α promoter showed high activity (Fig. 2C). The analysis of mean fluorescence index (MFI) by flow cytometry further supported the visual fluorescence results (Fig. 2D). These analyses confirmed the megakaryocytic specificity of Gp and ITGA promoters, and the endothelial specificity of VEC and KDR promoters.

**Fig. 3:**
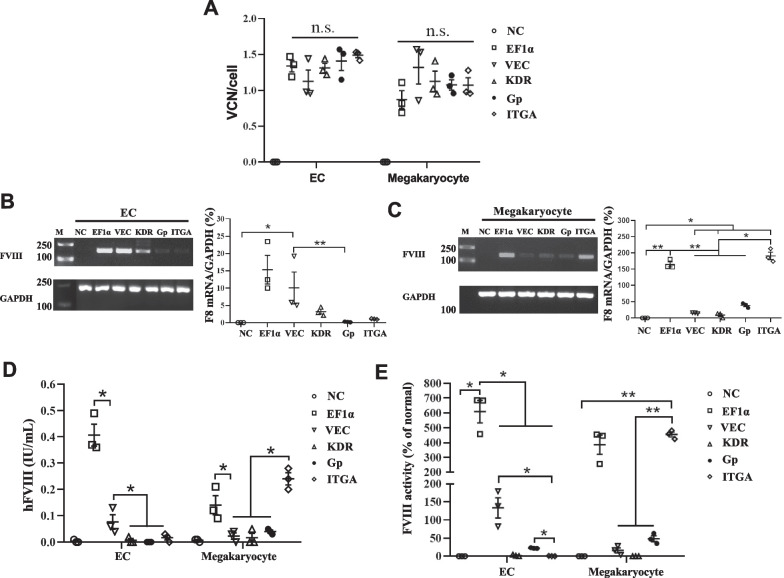
In vitro analyses of LV-*F8BDD* expression under different promoters in ECs and megakaryocytes. **A** Illustration of transduction efficiencies (VCN/cell) of the different LV promoter constructs (n = 3). **B**, **C** mRNA levels as percentages (%) of F8/GAPDH mRNA in ECs (**B**) and megakaryocytes (**C**) determined by gel electrophoresis (left) and RT-qPCR (right) (n = 3). **D** Protein concentrates detected using a human FVIII ELISA kit in ECs and megakaryocytes (n = 3). **E** FVIII activities determined by FVIII: C chromogenic assay in ECs and megakaryocytes (n = 3). The differences in characteristics between groups were analyzed using the one way Welch ANOVA tests with Games-Howell post hoc tests (**A**–**C**, **E**) and Kruskal–Wallis tests (**D**); *p < 0.05, **p < 0.01, ****p < 0.0001, n.s., no significant difference

We next examined tissue-specific *F8BDD* expression of the different LV promoter constructs in EA-hy926 and DAMI cells under the same MOI. The transduced cells contained similar VCNs as verified by qPCR (Fig. 3A). Based on RNA analysis, we found that the LV-EF1α-*F8BDD* expressed the highest amount of *F8* RNA in ECs, and the LV-VEC-*F8BDD* expressed significantly higher level of *F8* RNA than LV-Gp-*F8BDD* expressed in ECs (p < 0.05, Fig. 3B). In megakaryocytes, both the LV-ITGA-*F8BDD* and LV-EF1α-*F8BDD* expressed significantly higher amount of *F8* RNA than the other tissue-specific promoters (p < 0.05 and p < 0.01, Fig. 3C).

The FVIII protein expression was analyzed based on ELISA (Fig. 3D) and intracellular immunofluorescence staining using anti-FVIII antibody (Additional file 1: Fig. S2). We detected high FVIII expression under the universal EF1α promoter in all cell types, and LV-ITGA-*F8BDD* expressed FVIII protein similar to the EF1α promoter in megakaryocytes (Fig. 3D). The LV-VEC-*F8BDD*, while showed higher FVIII expression than the other tissue-specific promoters in EC (p < 0.05), its activities in both cell types were significantly lower than the EF1α promoter (~ 4–6 fold, p < 0.05). In addition, LV-ITGA-*F8BDD* showed higher FVIII expression than the other tissue-specific promoters in megakaryocytes (p < 0.05, Fig. 3D).

We further evaluated FVIII function based on the chromogenic assay. In EA-hy926 cells, we detected FVIII activities in the therapeutic range for both EF1α and VEC LVs, approximately 5 folds and 1.5 folds above the normal range, respectively (% of normal level, p < 0.05, Fig. 3E), while the other promoters did not show detectable FVIII activities in ECs. In megakaryocytes (DAMI), we detected high FVIII activity for both LV-EF1α-*F8BDD* and LV-ITGA-*F8BDD*, consistent with the RNA and protein analysis results, both at 4 folds above the normal level (p < 0.01), while the LV-Gp-*F8BDD* showed low FVIII activity (0.5 fold of the normal level). However, we did not detect FVIII activity for the KDR promoters in both EA-hy926 and DAMI cells (Fig. 3E), conceivably because of the short synthetic promoter that may not restrict it to an early-stage endothelial specificity.

### Enhanced iv LV gene delivery of tissue-specific reporter gene

**Fig. 4:**
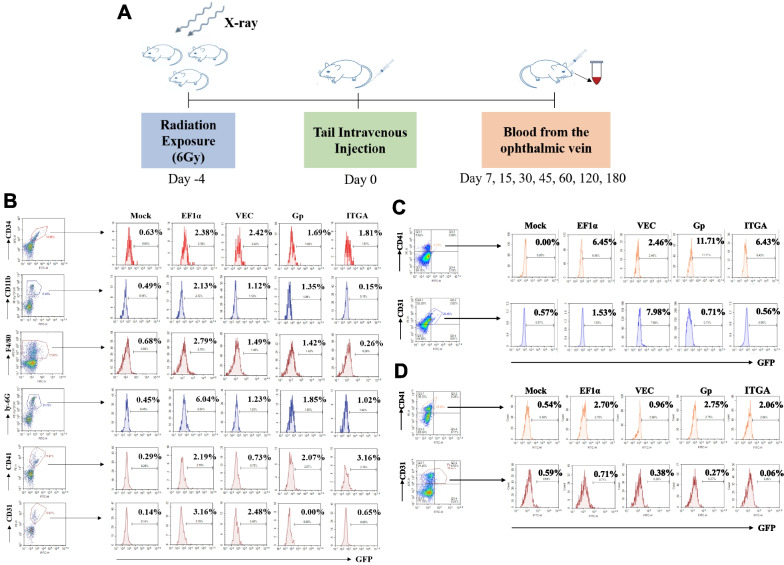
Enhanced iv LV-*mWasabi* gene transfer in mice after non-myeloablative immune suppression. **A** Illustration of tail vein injection of LV-*mWasabi* into WT mice and LV-*F8BDD* into *F8*^*null*^ mice pretreated with non-myeloablative radiation (6 Gy). The WT mice received tail vein iv injection of LV-*mWasabi* under the different promoters, EF1α, VEC, Gp and ITGA, at 1 × 10^7^ TU per mouse or 100 μL PBS per mouse as mock control. The blood was collected on Day 7, 15, 30, 45, 60, 120 and 180 after injection. **B–D** LV-GFP expression analysis by flow cytometry in BM, liver and spleen on day 30. The BM cells (**B**) were analyzed using antibodies for the different lineage-specific markers including CD34 for hematopoietic stem/progenitor cells, CD11b for monocytes/macrophages or DCs, Ly-6G for granulocytes and F4/80 for mature macrophages. In addition, the BM, liver (**C**) and spleen cells (**D**) were analyzed using Abs to CD41, a megakaryotic marker and CD31, an early endothelial marker

A potential limitation in gene therapy is the incidence of FVIII-specific immune response, which could decrease gene therapy efficacy due to the loss of F8 gene transduced cells (Nayak and Herzog 2010; Annoni et al. 2013). To investigate LV gene transfer in vivo, we first attempted direct iv injection in mice. Repeated LV iv injections failed to detect LV green fluorescence reporter gene expression or integrated LV genomes in the blood cells (Additional file 1: Fig. S2). We hypothesized that immunosuppression was necessary before HA gene therapy. To improve iv gene transfer and enhance LV transduction efficiency, we explored non-myeloablative conditioning prior to LV injection. WT mice were immunosuppressed with non-lethal dose radiation (600 cGy) 4 days prior to LV iv injection. The KDR promoter was not further tested because it did not showed FVIII protein expression and function in ECs in the in vitro assays. The mice were iv injected with LV-EF1α-*mWasabi*, LV-VEC-*mWasabi*, LV-Gp-*mWasabi* and LV-ITGA-*mWasabi* (3 × 10^8^ TU per animal; n = 3), or sterile PBS (mock, 200 μL per animal; n = 3) as illustrated in Fig. 4A. LV GFP expression was evaluated in the bone marrow together with lineage-specific marker staining including CD34 (HSCs), CD11b (primary monocytes/macrophages), F4/80 (mature macrophages) and Ly-6G (granulocytes) on day 30 (Fig. 4B). We found that the different promoters in LVs showed similar low levels of GFP expression in the CD34 + cells (1–2%). The LV-EF1α-*mWasabi* showed the highest expression in monocytes/macrophages (2.13–2.79%) and granulocytes (6.04%). The transgene expression was also examined in liver and spleen by flow cytometry co-immunostained for megakaryocyte (CD41) and endothelial (CD31) cell markers on day 30 after injection (liver in Fig. 4C, and spleen in Fig. 4D). The results showed high GFP expression in CD41-positive megakaryocytes from liver and spleen with LV-Gp-*mWasabi* (11.71% and 2.75%, respectively) and LV-ITGA-*mWasabi* (6.43% and 2.06%, respectively), and high GFP expression in CD31-positive ECs from liver and spleen with LV-VEC-*mWasabi* (7.98% and 0.38%, respectively).

### Functional and phenotypic correction of hemophilia A deficiency after iv LV injection in *F8*^*null*^ mice

**Fig. 5:**
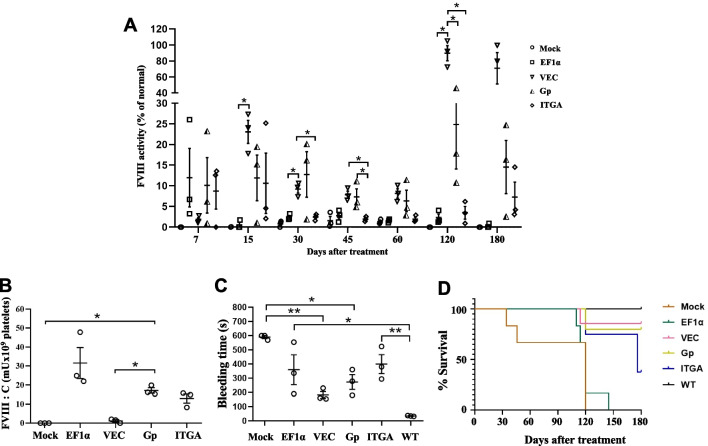
Prolonged FVIII functional and phenotype correction in *F8*^*null*^ mice after tail vein injection LV-VEC-*F8BDD*. The *F8*^*null*^ mice were treated with non-myeloablative radiation and given an iv injection of LV-*F8BDD* under the control of EF1α, VEC, Gp and ITGA promoters (1 × 10^7^ TU per animal) or PBS (100 μL per animal) mock control. **A** The kinetics of FVIII activities in plasma examined by the chromogenic assay on days 7, 15, 30, 45, 60, 120 and 180 (n = 3). **B** The FVIII acticities in 1 × 10^9^ platelets based on the chromogenic assay at day 60 (n = 3). **C** The tail bleeding time analysis recorded at day 120. The time required to stop bleeding into the collection tubes containing saline solution was recorded and plotted (n = 3). **D** The percentage survival curves of mice after LV injection for up to 180 days. The tail clipping experiment was carried out at day 120. The differences in characteristics between groups were analyzed using the one way Welch ANOVA tests with Turkey post hoc tests (**A**, **C**) or Games-Howell post hoc tests (A and B); *p < 0.05, **p < 0.01, ****p < 0.0001

To investigate the in vivo LV FVIII activities, *F8*^*null*^ mice were conditionally irradiated (600 cGy) and iv injected with 1 × 10^7^ TU of the different human *F8BDD* LVs (EF1α, VEC, Gp and ITGA), or PBS. Firstly, FVIII activities in the plasma were examined by the chromogenic assay on day 7, 15, 30, 45, 60, 120 and 180. The results showed that the LV-VEC-*F8BDD* and LV-Gp-*F8BDD* treated mice maintained a stable FVIII function (8%-25%) in 60 days, which increased to 80% in the LV-VEC-*F8BDD* treated mice and 15% in the LV-Gp-*F8BDD* treated mice after 120 days (p < 0.05). On the other hand, the LV-EF1α-*F8BDD* treated mice exhibited gradually reduced FVIII activities (below 3%) after 30 days, and the plasma FVIII activity in LV-ITGA-*F8BDD* treated mice was always less than 10% (Fig. 5A). Consistent with results in Fig. 5A, the aPTT analyses of plasma FVIII activity showed that all groups displayed function on day 7 after treatment. After 180 days, the LV-VEC-F8BDD and LV-Gp-F8BDD groups exhibited shorter clotting time, and the LV-VEC-F8BDD group exhibited faster clotting time than the LV-ITGA-F8BDD treated group (p < 0.05). The LV-Gp-F8BDD and LV-ITGA-F8BDD treated mice displayed activities significantly different from the WT mice, whereas the Mock and LV-EF-F8BDD treated mice died, likely due to poor clotting function (Additional file 1: Fig. S3). We further examined the FVIII protein levels in the platelets, and found the highest levels in the LV-EF1α-*F8BDD* treated mice, and that the LV-Gp-*F8BDD* treated mice exhibited higher FVIII levels than the LV-VEC-*F8BDD* treated mice on day 60 (p < 0.05) (Fig. 5B).

To examine bleeding phenotype, we subjected the *F8*^*null*^ mice to the bleeding diathesis test by tail clipping 4 months after LV injection. The results showed reduced bleeding in the LV-VEC-*F8BDD* group (p < 0.01) and the LV-Gp-*F8BDD* group (p < 0.05), as compared with the mock-treated *F8*^*null*^ mice, albeit, not as fully active as the WT mice in repeated tests (Fig. 5C). Both the LV-EF1α-*F8BDD* and LV-ITGA-*F8BDD* treated mice showed marginal improvement in the bleeding test. These results confirmed that the clotting function was restored in the LV-VEC-*F8BDD* and LV-Gp-*F8BDD* treated mice. For survival evaluation of tail clipping, six of seven LV-VEC-*F8BDD* treated mice, two of four LV-ITGA-*F8BDD* treated mice, four of five LV-Gp-*F8BDD* treated mice, and all of the WT mice survived the tail clip-induced bleeding test at 180 days, yet in contrast, all six LV-EF1α-*F8BDD* treated mice and the untreated control *F8*^*null*^ mice died in 120–140 days (Fig. 5D).

### Reduced FVIII inhibitory response in HA mice iv-treated with LV-VEC-*F8BDD*

**Fig. 6:**
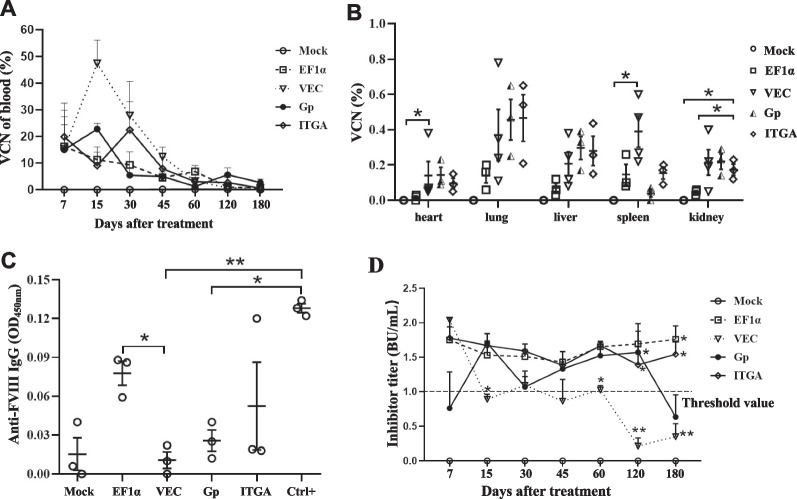
The kinetics of VCN and FVIII inhibitor formation in LV-*F8BDD* iv injected *F8*^*null*^ mice. **A** The VCNs in blood cells were detected by genomic DNA qPCR in LV-treated *F8*^*null*^ mice over time on days 7, 15, 30, 45, 60, 120 and 180 (n = 3). **B** The VCNs in the mouse organs including heart, lung, liver, spleen and kidney of the LV-treated *F8*^*null*^ mice on day 120 (n = 3). **C** Determination of anti-FVIII IgG levels by ELISA in the LV-treated *F8*^*null*^ mice. The plasma of LV-treated mice was collected 60 days after iv injection and diluted at 1:200 to determine anti-FVIII IgG levels (n = 3). **D** Analysis of inhibitor titer kinetics using plasma from LV-trated *F8*^*null*^ mice over time on days 7, 15, 30, 45, 60, 120 and 180 (n = 3). The inhibitor titer was determined based on a modified Bethesda unit (BU) assay; * D15 and D120 of VEC vs. Gp, * D60 of EF1α vs. VEC, * D60, D120 and D180 of VEC vs. ITGA, ** D120 and D180 of EF1α vs. VEC, * D180 of EF1α vs. Gp. The differences in characteristics between groups were analyzed using the one way Welch ANOVA tests with Turkey post hoc tests (**D**) or Games-Howell post hoc tests (**B**–**D**); *p < 0.05, **p < 0.01, ***p < 0.001

The FVIII-specific inhibitory response is a major limitation in HA gene therapy. To examine the anti-FVIII activities, we measured FVIII-specific inhibitor formation in the LV iv injected mice. To monitor gene transfer efficiencies, the VCNs in the blood of the treated mice were monitored by qPCR. The VCN kinetics showed peak 48%, 25% and 10% of gene-modified cells in the VEC, Gp and ITGA LV-treated mice, respectively, on day 15 after treatment, which gradually reduced to ~ 1% after 180 days (Fig. 6A). The VCN in blood was detected from peak 12% to nearly 0% in the EF1α LV-treated mice in 180 days (Fig. 6A). The VCNs in different organs (heart, lung, liver, spleen and kidney) were also examined by qPCR upon sacrifice on day 120. We found that both heart and kidney contained low VCNs (< 0.22%) as compared with lung (0.37–0.56%) in all of the treated mice. The VCNs in heart and spleen of the LV-VEC-*F8BDD* treated mice were consistently higher than the untreated mice (p < 0.05); and VCNs in kidney of the LV-ITGA-*F8BDD* treated mice were higher than the LV-EF1α-*F8BDD*-treated and the untreated mice (p < 0.05) (Fig. 6B).

The FVIII inhibitor activities in the plasma after LV injection were assessed based on FVIII IgG and Bethesda assays. At 60 days after iv injection, we detected low anti-FVIII IgG response in the LV-VEC-*F8BDD* treated mice, whereas the LV-EF1α-*F8BDD* treated mice exhibited high IgG response (p < 0.05) (Fig. 6C). On the other hand, both LV-VEC-*F8BDD* and LV-Gp-*F8BDD* treated mice exhibited a trend of decreasing inhibitor titer (from 2 to 0.4 BU/mL) by the Bethesda assay 180 days after iv LV injection, suggesting increasing FVIII activities with time, whereas mice treated with the other LVs showed consistent high inhibitor titers with time (1.5–2 BU/mL, Fig. 6D).

## Discussion

The liver has been implicated as the major FVIII protein production organ, because liver transplantation is able to cure HA in canine models and humans (Maestro et al. 2021). Previous studies have demonstrated the importance of hepatocytes as the main physiologic source of FVIII. Moreover, hepatocyte-specific transgene expression may induce tolerance, reducing the risk for immune reaction against the *F8* transgene, which addresses the current limitations in FVIII therapies for HA (High et al. 2014). More recently, the preponderance of evidence implicates that FVIII protein is systhesized by liver sinusoids endothelial cells (LSECs) and by extension, endothelial cells in other tissues as well, which may explain the observations of FVIII production in extrahepatic vascularized tissues such as kidney, spleen, and lung (Herzog 2015; Jacquemin et al. 2006; El-Maarri et al. 2020), and to a lesser extent by hepatocytes in humans and mice (Follenzi et al. 2008; Hayakawa et al. 2021; Kumaran et al. 2005; Shahan et al. 2014; Fahs et al. 2014; Everett et al. 2014). This is further complicated by FVIII stabilization through interaction with von Willebrand factor (vWF) (Gong et al. 2022; Montgomery and Shi 2012), and the latter is known to be produced by endothelial cells, platelets, and megakaryocytes (Pablo-Moreno et al. 2022). Thus, the combination of expression in an unnatural cell type and the lack of vWF synthesis in hepatocytes may explain the difficulties that have been encountered in inducing hepatic expression of FVIII protien. All of the above suggest that FVIII protein synthesis in LSECs and tissue ECs might be preferred for therapeutic FVIII expression.

Infusions of therapeutic cells including LSECs and myeloid cells have demonstrated FVIII protein secretion and decreased bleeding in *F8*^*null*^ mice (Follenzi et al. 2008; Follenzi et al. 2012; Matsui 2012). Here we investigated targeted FVIII expression in ECs and megakaryocytes using LVs containing endothelial-specific (VEC and KDR) or megakaryocyte-specific (ITGA and Gp) promoters. The tissue-specific functional FVIII expression was illustrated both in vitro and in vivo. Merlin et al. have previously established a LV construct containing the endothelial-specific murine VEC promoter together with miRT122 and miRT142-3p to prevent expression in hepatocytes and hematopoietic cells, respectively, and reported findings similar to our iv LV gene therapy but used higher amount of LVs, 5 × 10^8^ total TU per mouse, as compared with 1 × 10^7^ TU per mouse in this study (Merlin et al. 2017). Such difference could be explained either by different measurement on LV TU or the non-myeloablasive immunosuppressive conditioning protocol applied in our study. Furthermore, endothelial-specific CD105-mediated cell entry using targeted LVs for systemic gene transfer into LSECs, although at a very low efficiency still, might be an alternative targeted HA gene therapy strategy (Vandendriessche and Chuah 2013; Abel et al. 2013).

A potential limitation in gene therapy is the incidence of FVIII-specific immune response, which may decrease gene therapy efficacy due to the loss of *F8* gene transduced cells (Nayak and Herzog 2010; Annoni et al. 2013). Miao et. al. have shown that dexamethasone and anti-CD8a antibody treatment can enhance LV transduction efficiency and suppress cytotoxic responses for LV gene therapy in *F8*^*null*^ mice. We applied myelosuppression by using low dose radiation, with the goal to obtain temporary immunosuppression to facilitate stable gene transfer. This non-myeloablative conditioning facilitated enhanced LV gene transfer efficiencies in vivo. We have also explored chemotherapy conditioning to facilitate LV gene transfer via iv injections in support of future clinical applications (manuscript in preparation).

For a long time, the main limitations in gene therapy is low transgenic efficiency and suboptimal funcitonal FVIII activities. There has not been direct comparison of a strong universal promoter versus tissue-specific promoter to drive FVIII expression in vivo. We established an improved iv LV delivery approach, illustrating high F8 expression under the EF1α promoter in differentiated myeloid cells as compared with the tissue-specific promoters in the *F8*^*null*^ mice (Fig. 4). Such ubiquitous FVIII protein expression under the universal promoter evidently induced an overt anti-FVIII immune response, which resulted in the ablation of the *F8* transgene with time (Figs. 5, 6). Importantly, the EC-specific FVIII protein expression under the VEC promoter demonstrated functional correction of HA phenotype requiring only very low level of ectopic FVIII expression. In addition, the LV-VEC-*F8BDD* also displayed increased LV packaging efficiency. As such, iv delivery of the EC-specific LV-VEC-*F8BDD* could substantially reduce the adverse immune risk as well as the high cost as compared with ex vivo HSCT-based gene therapy for HA.

Whilst megakaryocytes in the hemopoisis system are important hemostasis components, and gene therapy through HSC modification or intraosseous injection could effectively target megakaryocytes, it is not as safe and convenient as the direct iv approach. Merlin et al. have designed targeted LVs based on VEC and myeloid-specific promoters plus miRNAs to restrict FVIII expression in the LSECs and myeloid cells, and reported effective LV gene therapy in HA mice without inhibitory immune response (Merlin et al. 2017). Whether the VEC promoter alone could achieve a full therapeutic effect in HA gene therapy, however, has not been investigated. Using the LV-VEC-*F8BDD*, we demonstrated efficient transgene delivery and EC-specific expression even at a tenfold less LV dose with low VCNs in blood cells (~ 1–2%), as well as in various organs (< 0.8%), yet still, exhibited full coagulation function (~ 100%, Fig. 5B). Further in vivo analyses of the LV-VEC-*F8BDD* treated mice illustrated a low FVIII inhibitory response with prolonged phenotype correction (Fig. 6).

## Conclusion

This study showed that a simple iv injection of LV-VEC-*F8BDD* could establish targeted ectopic FVIII expression in ECs with restored coagulation function and low inhibitory immunogenicity in the *F8*^*null*^ mice, which has a great potential to be translated in future clinical applications.

## Supplementary Information

**Additional** **file** **1:** **Figure** **S1.** The human FVIII protein expression in LV-transduced ECs. The hF8 protein was detected in ECs transduced with LV-*F8BDD* under different promoters by intracellular staining using anti-FVIII Ab conjugated with FITC under a fluorescent microscope; scale bars, 20 μm. **Figure** **S2.** The green fluorescence expression in WT mice after LV-*mWasabi* tail vein injection without non-myeloablative treatment. The blood mononuclear cells from PBSor LV-*mWasabi*treated WT mice were analyzed by flow cytometry seven days after tail vein LV injection. **Figure** **S3.** The FVIII activity assessed by aPTT. The aPTT assay results which confirmed FVIII activities and phenotypic correction in the *F8*^*null*^ mice; 7 days after treatment, and those survived the tail clipping test on day 180 after treatment.

**Additional** **file** **2**: **Table** **S1**. The transduction efficiencies of LV-*mWasabi* in different cell types. **Table** **S2**. The GFP levels in different murine blood cell subset analyzed by flow cytometry at Day 30. **Table** **S3**. The numerical data of Fig. 2A. **Table** **S4**. The numerical data of Fig. 2B. **Table** **S5**. The numerical data of Figure 2D. **Table** **S6**. The numerical data of Fig. 3A. **Table** **S7**. The numerical data of Fig. 3B, C. **Table** **S8**. The numerical data of Fig. 3D. **Table** **S9**. The numerical data of Fig. 3E. **Table** **S10**. The numerical data of Fig. 5A. **Table** **S11**. The numerical data of Fig. 5B. **Table** **S12**. The numerical data of Fig. 5C. **Table** **S13**. The numerical data of Fig. 6A. **Table** **S14**. The numerical data of Fig. 6B. **Table** **S15**. The numerical data of Fig. 6C. **Table** **S16**. The numerical data of Fig. 6D.

## Data Availability

All raw data used for figure generation in this study can be obtained by contacting the corresponding author.

## Abbreviations

AAV: Adeno-associated virus vector
Abs: Antibodies
*F8BDD*: B-domain deleted human *F8* gene
BM: Bone marrow
ECs: Endothelial cells
*F8*: Factor FVIII
*F8*^*null*^ mice: *F8* Knockout mice
gDNA: Genomic DNA
GFP: Green fluorescence protein
Gp: Gp1bα
HA: Hemophilia A
iv: Intravenous
HSCs: Hematopoietic stem cells
*hF8*: Human *F8* gene
LSECs: Liver sinusoids endothelial cells
LV: Lentiviral vectors/lentivirus
MFI: Mean fluorescence index
MOI: Multiplicities of infection
PBMCs: Peripheral blood mononuclear cells
PRT: Protein replacement therapy
qPCR: Quantitative PCR
VEGF: Vascular endothelial growth factor
VCN: Vector copy number
WT: Wild-type
