# Supplementary figures and images for: Improved intravenous lentiviral gene therapy based on endothelial-specific promoter-driven factor VIII expression for hemophilia A
Source: Mol Med. 2023 Jun 12;29:74. doi: 10.1186/s10020-023-00680-z (PMC10262495; doi:10.1186/s10020-023-00680-z)

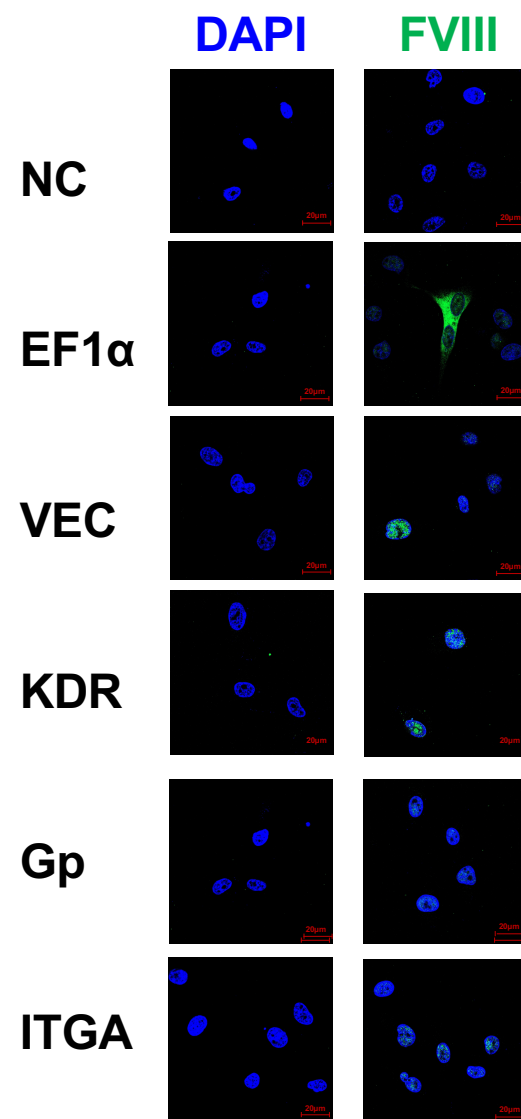

Fig. S1

**A**

**Mock**

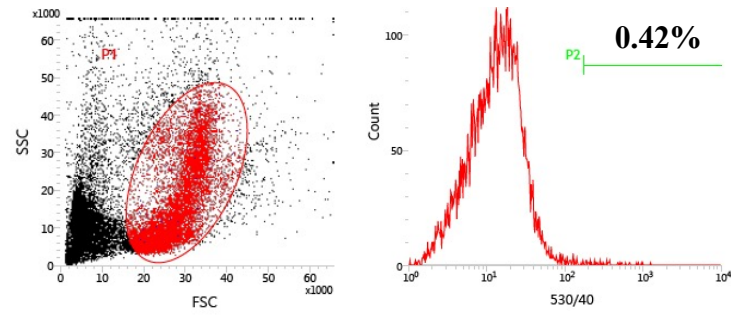

**B**

**iv**  
***LV.mWasabi***

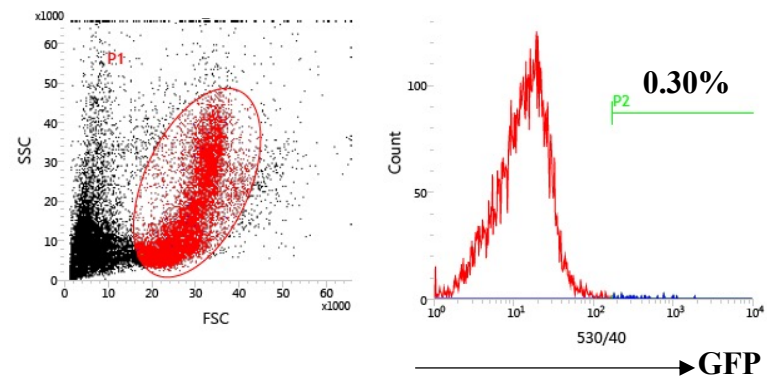

**Fig. S2**

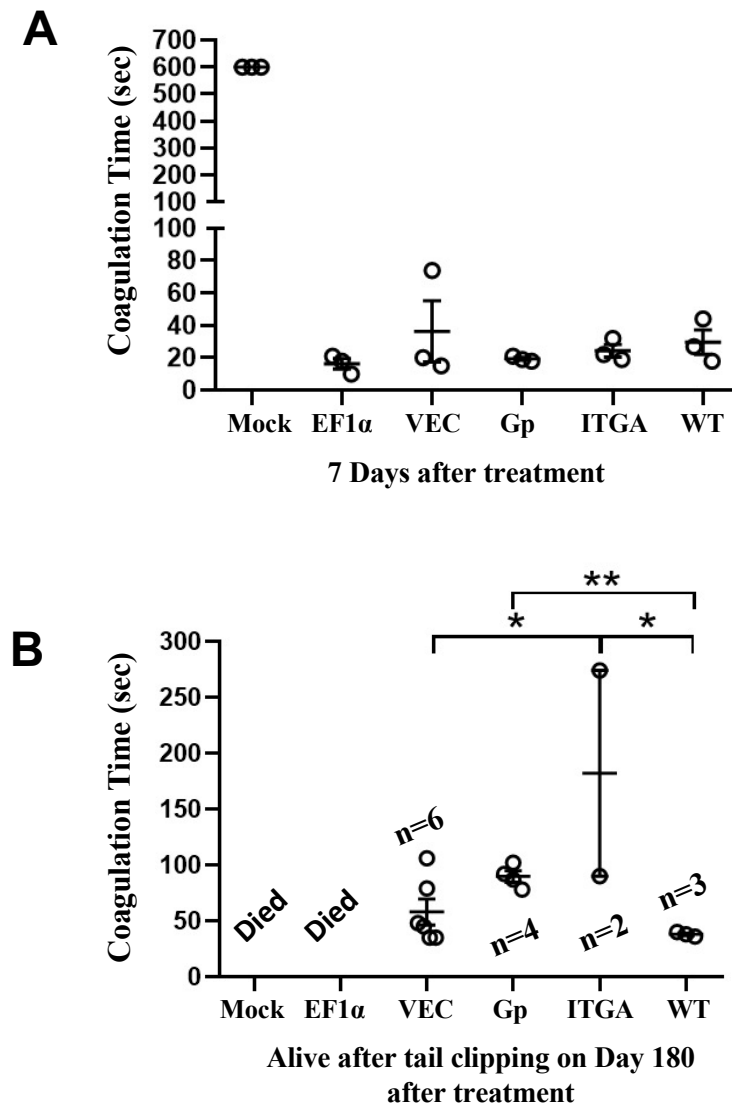

Fig. S3

Supplement: Supplementary file 1 — Additional file 1: Figure S1. The human FVIII protein expression in LV-transduced ECs. The hF8 protein was detected in ECs transduced with LV-F8BDD under different promoters by intracellular staining using anti-FVIII Ab conjugated with FITC under a fluorescent microscope; scale bars, 20 μm. Figure S2. The green fluorescence expression in WT mice after LV-mWasabi tail vein injection without non-myeloablative treatment. The blood mononuclear cells from PBSor LV-mWasabitreated WT mice were analyzed by flow cytometry seven days after tail vein LV injection. Figure S3. The FVIII activity assessed by aPTT. The aPTT assay results which confirmed FVIII activities and phenotypic correction in the F8null mice; 7 days after treatment, and those survived the tail clipping test on day 180 after treatment. [file 10020_2023_680_MOESM1_ESM.pdf]
