# Supplementary material for: Improved intravenous lentiviral gene therapy based on endothelial-specific promoter-driven factor VIII expression for hemophilia A
Source: Mol Med. 2023 Jun 12;29:74. doi: 10.1186/s10020-023-00680-z (PMC10262495; doi:10.1186/s10020-023-00680-z)
Supplement: Supplementary file 2 — Additional file 2: Table S1. The transduction efficiencies of LV-mWasabi in different cell types. Table S2. The GFP levels in different murine blood cell subset analyzed by flow cytometry at Day 30. Table S3. The numerical data of Fig. 2A. Table S4. The numerical data of Fig. 2B. Table S5. The numerical data of Figure 2D. Table S6. The numerical data of Fig. 3A. Table S7. The numerical data of Fig. 3B, C. Table S8. The numerical data of Fig. 3D. Table S9. The numerical data of Fig. 3E. Table S10. The numerical data of Fig. 5A. Table S11. The numerical data of Fig. 5B. Table S12. The numerical data of Fig. 5C. Table S13. The numerical data of Fig. 6A. Table S14. The numerical data of Fig. 6B. Table S15. The numerical data of Fig. 6C. Table S16. The numerical data of Fig. 6D. [file 10020_2023_680_MOESM2_ESM.docx]

**Table S1. The transduction efficiencies of LV-*mWasabi* in different cell types.**

| **LV VCN (% per cell)** | **ECs** | **Megakaryocytes** | **Myeloid cells** | **Lymphocytes** |
| --- | --- | --- | --- | --- |
| **NC** | 0.00 | 0.00 | 0.00 | 0.00 |
| **EF1α** | 144.75 | 520.97 | 80.96 | 105.92 |
| **VEC** | 105.25 | 484.56 | 95.63 | 89.65 |
| **KDR** | 85.43 | 451.82 | 92.35 | 98.44 |
| **Gp** | 95.42 | 585.71 | 88.66 | 95.70 |
| **ITGA** | 83.54 | 445.99 | 75.18 | 113.75 |

**Table S2. The GFP levels in different murine blood cell subset analyzed by flow cytometry at Day 30.**

| **GFP %** | **Monocytes** | **Lymphocytes** | **Granulocytes** | **Total** |
| --- | --- | --- | --- | --- |
| **Mock** | 0.50±0.50 | 0.65±0.18 | 0.69±0.15 | 0.38±0.05 |
| **EF1α** | 0.77±0.36 | 4.32±1.43 | 4.22±0.97 | 0.40±0.04 |
| **VEC** | 1.33±1.78 | 0.52±0.07 | 0.42±0.07 | 0.35±0.09 |
| **Gp** | 0.35±0.10 | 0.63±0.13 | 2.72±1.77 | 0.52±0.14 |
| **ITGA** | 1.78±0.15 | 0.40±0.05 | 2.88±1.19 | 0.26±0.07 |

**Table S3. The numerical data of Figure 2A.**

| **LV titer (10^9^ TU/mL)** | **EF1α**  **(n=4)** | **VEC**  **(n=4)** | **KDR**  **(n=4)** | **Gp**  **(n=4)** | **ITGA**  **(n=4)** |
| --- | --- | --- | --- | --- | --- |
| ***mWasabi*** | 1.2 | 3 | 5.5 | 7.7 | 4.2 |
|  | 4.3 | 6.3 | 2.7 | 5.3 | 4.2 |
|  | 5.3 | 6.6 | 2.4 | 3 | 5 |
|  | 1.7 | 4.6 | 1.4 | 4.1 | 2.6 |

**Table S4. The numerical data of Figure 2B.**

| **LV titer (10^9^ TU/mL)** | **EF1α**  **(n=7)** | **VEC**  **(n=3)** | **KDR**  **(n=3)** | **Gp**  **(n=4)** | **ITGA**  **(n=4)** |
| --- | --- | --- | --- | --- | --- |
| ***F8BDD*** | 5 | 160 | 3 | 3 | 57 |
|  | 8 | 38 | 1 | 11 | 15 |
|  | 20 | 170 | 2 | 2 | 7 |
|  | 29 | / | / | 9 | 95 |
|  | 1 | / | / | / | / |
|  | 6 | / | / | / | / |
|  | 4 | / | / | / | // |

**Table S5. The numerical data of Figure 2D.**

| **MFI** | **ECs**  **(n=3)** | | | **Megakaryocytes**  **(n=3)** | | | **Myeloid cells**  **(n=3)** | | | **Lymphocytes**  **(n=3)** | | |
| --- | --- | --- | --- | --- | --- | --- | --- | --- | --- | --- | --- | --- |
| **NC** | 0.11 | 0.10 | 0.11 | 0.03 | 0.02 | 0.03 | 0.36 | 0.38 | 0.37 | 0.34 | 0.38 | 0.63 |
| **EF1α** | 7.13 | 6.88 | 7.10 | 29.60 | 27.17 | 27.58 | 18.62 | 13.51 | 17.50 | 12.95 | 11.75 | 7.60 |
| **VEC** | 0.53 | 0.48 | 0.53 | 0.34 | 0.52 | 0.45 | 0.71 | 0.82 | 1.20 | 0.78 | 1.30 | 1.10 |
| **KDR** | 0.60 | 0.57 | 0.61 | 1.49 | 1.27 | 1.30 | 0.84 | 0.84 | 0.65 | 1.45 | 1.24 | 0.68 |
| **Gp** | 0.29 | 0.24 | 0.29 | 2.30 | 2.13 | 2.16 | 0.96 | 0.57 | 0.98 | 1.19 | 1.57 | 0.86 |
| **ITGA** | 0.35 | 0.33 | 0.36 | 6.60 | 5.75 | 5.83 | 0.94 | 0.76 | 0.68 | 1.15 | 1.39 | 0.91 |

**Table S6. The numerical data of Figure 3A.**

| **LV VCN**  **(per cell)** | **ECs (n=3)** | | | **Megakaryocytes (n=3)** | | |
| --- | --- | --- | --- | --- | --- | --- |
| **NC** | 0.00 | 0.00 | 0.00 | 0.00 | 0.00 | 0.00 |
| **EF1α** | 1.36 | 1.19 | 1.47 | 0.82 | 0.69 | 1.11 |
| **VEC** | 0.98 | 0.96 | 1.44 | 1.53 | 0.86 | 1.57 |
| **KDR** | 1.28 | 1.22 | 1.44 | 1.02 | 0.95 | 1.41 |
| **Gp** | 1.15 | 1.57 | 1.51 | 1.21 | 1.06 | 0.96 |
| **ITGA** | 1.53 | 1.42 | 1.52 | 0.96 | 0.98 | 1.28 |

**Table S7. The numerical data of Figure 3B and 3C.**

| **F8 mRNA /GAPDH(%)** | **ECs** **(n=3)** | | | **Megakaryocytes (n=3)** | | |
| --- | --- | --- | --- | --- | --- | --- |
| **NC** | 0.00 | 0.00 | 0.00 | 0.00 | 0.00 | 0.00 |
| **EF1α** | 10.01 | 12.38 | 23.45 | 157.27 | 156.34 | 180.03 |
| **VEC** | 5.06 | 5.87 | 19.19 | 13.35 | 15.89 | 15.51 |
| **KDR** | 2.96 | 4.52 | 2.15 | 13.16 | 13.26 | 0.00 |
| **Gp** | 0.1 | 0.22 | 0.32 | 40.66 | 44.95 | 30.8 |
| **ITGA** | 0.93 | 1.05 | 1.15 | 185.26 | 173.8 | 212.85 |

**Table S8. The numerical data of Figure 3D.**

| **hFVIII (IU/mL)** | **ECs (n=3)** | | | **Megakaryocytes (n=3)** | | |
| --- | --- | --- | --- | --- | --- | --- |
| **NC** | 0.00 | 0.01 | 0.00 | 0.01 | 0.01 | 0.00 |
| **EF1α** | 0.49 | 0.36 | 0.37 | 0.12 | 0.09 | 0.21 |
| **VEC** | 0.13 | 0.04 | 0.06 | 0.04 | 0.03 | 0.00 |
| **KDR** | 0.02 | 0.00 | 0.00 | 0.00 | 0.05 | 0.00 |
| **Gp** | 0.00 | 0.00 | 0.00 | 0.04 | 0.05 | 0.03 |
| **ITGA** | 0.02 | 0.00 | 0.03 | 0.24 | 0.2 | 0.28 |

**Table S9. The numerical data of Figure 3E.**

| **FVIII activity (% of normal)** | **ECs (n=3)** | | | **Megakaryocytes (n=3)** | | |
| --- | --- | --- | --- | --- | --- | --- |
| **NC** | 0.00 | 0.10 | 0.00 | 0.00 | 0.10 | 0.03 |
| **EF1α** | 683.40 | 685.50 | 457.50 | 445.75 | 454.50 | 255.67 |
| **VEC** | 138.70 | 179.70 | 82.33 | 16.58 | 3.67 | 28.17 |
| **KDR** | 4.40 | 1.50 | 0.43 | 0.00 | 0.00 | 0.00 |
| **Gp** | 21.80 | 23.60 | 21.50 | 63.25 | 47.00 | 35.67 |
| **ITGA** | 0.90 | 0.00 | 0.00 | 482.00 | 458.67 | 424.00 |

**Table S10. The numerical data of Figure 5A.**

| **Days after treatment** | **Mock (n=3)** | | | **EF1α (n=3)** | | | **VEC (n=3)** | | | **Gp (n=3)** | | | **ITGA (n=3)** | | |
| --- | --- | --- | --- | --- | --- | --- | --- | --- | --- | --- | --- | --- | --- | --- | --- |
| **7** | 0.00 | 0.00 | 0.00 | 6.66 | 26.00 | 3.21 | 2.72 | 1.08 | 1.41 | 6.16 | 0.92 | 23.21 | 0.00 | 12.56 | 13.54 |
| **15** | 0.00 | 0.00 | 0.10 | 0.00 | 0.00 | 1.74 | 17.80 | 27.31 | 24.03 | 1.08 | 19.44 | 15.18 | 25.18 | 2.07 | 4.52 |
| **30** | 1.41 | 1.08 | 0.00 | 2.07 | 1.9 | 3.21 | 10.59 | 9.61 | 7.31 | 1.90 | 16.16 | 20.10 | 2.56 | 1.57 | 3.05 |
| **45** | 3.54 | 0.92 | 0.26 | 1.25 | 2.89 | 4.03 | 6.49 | 7.48 | 9.44 | 6.00 | 4.85 | 11.08 | 1.41 | 1.90 | 2.56 |
| **60** | 1.9 | 1.25 | 0.92 | 1.57 | 1.08 | 1.90 | 8.13 | 6.16 | 10.10 | 4.69 | 2.89 | 11.41 | 1.57 | 2.89 | 1.41 |
| **120** | 0.00 | 0.00 | 0.00 | 1.57 | 1.25 | 4.03 | 92.07 | 105.02 | 72.39 | 17.80 | 10.75 | 46.00 | 6.16 | 0.92 | 3.38 |
| **180** | 0.00 | 0.00 | 0.00 | 0.92 | 0.10 | 0.00 | 33.21 | 99.61 | 79.77 | 16.33 | 24.69 | 2.56 | 14.52 | 3.05 | 4.20 |

**Table S11. The numerical data of Figure 5B.**

| **FVIII:C (mUx10^9^ platlets)** | **Mock (n=3)** | | | **EF1α (n=3)** | | | **VEC (n=3)** | | | **Gp (n=3)** | | | **ITGA (n=3)** | | |
| --- | --- | --- | --- | --- | --- | --- | --- | --- | --- | --- | --- | --- | --- | --- | --- |
|  | 0.00 | 0.00 | 0.00 | 24.92 | 22.00 | 47.83 | 0.00 | 2.00 | 1.58 | 19.92 | 16.17 | 15.33 | 15.75 | 8.25 | 14.92 |

**Table S12. The numerical data of Figure 5C.**

| **Bleeding time (s)** | |
| --- | --- |
| **Mock (n=3)** | 600 |
|  | 569 |
|  | 600 |
| **EF1α(n=3)** | 336 |
|  | 190 |
|  | 554 |
| **VEC (n=3)** | 161 |
|  | 230 |
|  | 155 |
| **Gp (n=3)** | 180 |
|  | 279 |
|  | 361 |
| **ITGA (n=3)** | 524 |
|  | 295 |
|  | 380 |
| **WT (n=3)** | 35 |
|  | 32 |
|  | 38 |

**Table S13. The numerical data of Figure 6A.**

| **Days after treatment** | **Mock (n=3)** | | | **EF1α (n=3)** | | | **VEC (n=3)** | | | **Gp (n=3)** | | | **ITGA (n=3)** | | |
| --- | --- | --- | --- | --- | --- | --- | --- | --- | --- | --- | --- | --- | --- | --- | --- |
| **7** | 0.00 | 0.00 | 0.00 | 48.72 | 0.00 | 0.00 | 39.12 | 0.00 | 6.91 | 0.00 | 12.38 | 32.49 | 0.00 | 31.35 | 28.32 |
| **15** | 0.00 | 0.00 | 0.00 | 20.60 | 5.34 | 7.94 | 54.78 | 30.11 | 57.42 | 19.14 | 26.47 | 22.71 | 17.49 | 9.69 | 0.00 |
| **30** | 0.00 | 0.00 | 0.00 | 18.85 | 6.48 | 2.60 | 30.35 | 4.45 | 48.73 | 0.00 | 15.45 | 0.90 | 43.28 | 14.51 | 9.70 |
| **45** | 0.00 | 0.43 | 0.00 | 2.69 | 2.23 | 8.77 | 6.74 | 11.23 | 19.11 | 4.72 | 9.75 | 0.43 | 0.52 | 15.42 | 7.90 |
| **60** | 0.59 | 0.00 | 0.00 | 9.12 | 9.08 | 2.30 | 5.17 | 3.03 | 1.53 | 1.25 | 2.09 | 0.35 | 0.31 | 8.36 | 0.38 |
| **120** | 0.57 | 0.00 | 0.00 | 0.06 | 1.86 | 1.09 | 0.05 | 0.41 | 1.76 | 0.83 | 9.58 | 6.50 | 0.48 | 6.38 | 1.23 |
| **180** | 0.00 | 0.00 | 0.00 | 1.69 | 0.00 | 0.28 | 0.00 | 1.11 | 0.96 | 3.87 | 0.00 | 4.10 | 0.25 | 0.00 | 1.49 |

**Table S14. The numerical data of Figure 6B.**

| **Days after treatment** | **Mock (n=3)** | | | **EF1α (n=3)** | | | **VEC (n=3)** | | | **Gp (n=3)** | | | **ITGA (n=3)** | | |
| --- | --- | --- | --- | --- | --- | --- | --- | --- | --- | --- | --- | --- | --- | --- | --- |
| **Heart** | 0.00 | 0.00 | 0.00 | 0.00 | 0.03 | 0.02 | 0.07 | 0.06 | 0.38 | 0.15 | 0.09 | 0.05 | 0.09 | 0.23 | 0.11 |
| **Lung** | 0.00 | 0.00 | 0.00 | 0.16 | 0.06 | 0.20 | 0.78 | 0.24 | 0.35 | 0.65 | 0.21 | 0.54 | 0.47 | 0.65 | 0.25 |
| **Liver** | 0.00 | 0.00 | 0.00 | 0.03 | 0.06 | 0.12 | 0.25 | 0.12 | 0.38 | 0.436 | 0.15 | 0.26 | 0.17 | 0.39 | 0.33 |
| **Spleen** | 0.00 | 0.00 | 0.00 | 0.26 | 0.10 | 0.08 | 0.60 | 0.27 | 0.47 | 0.20 | 0.17 | 0.09 | 0.00 | 0.07 | 0.05 |
| **Kidney** | 0.00 | 0.00 | 0.00 | 0.03 | 0.05 | 0.06 | 0.40 | 0.19 | 0.22 | 0.17 | 0.23 | 0.12 | 0.14 | 0.23 | 0.29 |

**Table S15. The numerical data of Figure 6C.**

| **Anti-FVIII IgG (OD_450nm_)** | |
| --- | --- |
| **Mock (n=3)** | 0.006 |
|  | 0.000 |
|  | 0.040 |
| **EF1α(n=3)** | 0.086 |
|  | 0.088 |
|  | 0.059 |
| **VEC (n=3)** | 0.010 |
|  | 0.000 |
|  | 0.022 |
| **Gp (n=3)** | 0.025 |
|  | 0.040 |
|  | 0.012 |
| **ITGA (n=3)** | 0.018 |
|  | 0.019 |
|  | 0.120 |
| **Ctrl^+^ (n=3)** | 0.122 |
|  | 0.134 |
|  | 0.128 |

**Table S16. The numerical data of Figure 6D.**

| **Days after treatment** | **Mock (n=3)** | | | **EF1α (n=3)** | | | **VEC (n=3)** | | | **Gp (n=3)** | | | **ITGA (n=3)** | | |
| --- | --- | --- | --- | --- | --- | --- | --- | --- | --- | --- | --- | --- | --- | --- | --- |
| **7** | 0.00 | 0.00 | 0.00 | 1.94 | 1.13 | 2.18 | 2.01 | 2.03 | 2.08 | 1.80 | 0.10 | 0.38 | 1.61 | 1.63 | 2.10 |
| **15** | 0.00 | 0.00 | 0.00 | 1.76 | 0.91 | 1.92 | 0.88 | 1.02 | 0.77 | 1.76 | 1.66 | 1.70 | 1.34 | 1.77 | 1.90 |
| **30** | 0.00 | 0.00 | 0.00 | 1.31 | 1.63 | 1.59 | 0.76 | 1.47 | 1.05 | 1.37 | 0.95 | 0.89 | 1.39 | 1.66 | 1.72 |
| **45** | 0.00 | 0.00 | 0.00 | 1.35 | 1.49 | 1.45 | 0.26 | 1.02 | 1.31 | 1.44 | 1.70 | 0.85 | 1.4 | 1.40 | 1.34 |
| **60** | 0.00 | 0.00 | 0.00 | 1.54 | 1.76 | 1.66 | 1.01 | 0.93 | 1.14 | 1.66 | 1.72 | 1.19 | 1.54 | 1.70 | 1.76 |
| **120** | 0.00 | 0.00 | 0.00 | 1.90 | 2.07 | 1.11 | 0.40 | 0.24 | 0.00 | 1.86 | 0.95 | 1.90 | 1.34 | 1.88 | 0.95 |
| **180** | 0.00 | 0.00 | 0.00 | 2.04 | 1.39 | 1.85 | 0.39 | 0.65 | 0.00 | 1.01 | 0.89 | 0.00 | 1.55 | 1.87 | 1.21 |
